# Heterogeneous disease progression and treatment response in a C3HeB/FeJ mouse model of tuberculosis

**DOI:** 10.1242/dmm.019513

**Published:** 2015-06-01

**Authors:** Jean-Philippe Lanoix, Anne J. Lenaerts, Eric L. Nuermberger

**Affiliations:** ^1^ Center for Tuberculosis Research, Department of Medicine, Johns Hopkins University School of Medicine, Baltimore, MD 21287, USA; ^2^ INSERM U1088, 80000 Amiens, France; ^3^ Mycobacteria Research Laboratories, Department of Microbiology, Immunology and Pathology, Colorado State University, Fort Collins, CO 80523, USA; ^4^ Department of International Health, Johns Hopkins Bloomberg School of Public Health, Baltimore, MD 21287, USA

**Keywords:** Tuberculosis, C3HeB/FeJ, Heterogeneity, pH, Pyrazinamide

## Abstract

Mice are the most commonly used species for non-clinical evaluations of drug efficacy against tuberculosis (TB). Unlike commonly used strains, C3HeB/FeJ mice develop caseous necrosis in the lung, which might alter the representation of drug efficacy in a way that is more like human TB. Because the development of such pathology requires time, we investigated the effect of infection incubation period on the activity of six drugs in C3HeB/FeJ and BALB/c mice. Mice were aerosol infected and held for 6, 10 or 14 weeks before receiving therapy with rifampin (RIF), rifapentine (RPT), pyrazinamide (PZA), linezolid (LZD), sutezolid (PNU) or metronidazole (MTZ) for 4-8 weeks. Outcomes included pathological assessments, pH measurements of liquefied caseum and assessment of colony-forming unit (CFU) counts from lung cultures. Remarkable heterogeneity in the timing and extent of disease progression was observed in C3HeB/FeJ mice, largely independent of incubation period. Likewise, drug efficacy in C3HeB/FeJ mice was not affected by incubation period. However, for PZA, LZD and PNU, dichotomous treatment effects correlating with the presence or absence of large caseous lesions were observed. In the case of PZA, its poor activity in the subset of C3HeB/FeJ mice with large caseous lesions might be explained by the pH of 7.36±0.09 measured in liquefied caseum. This study highlights the potential value of C3HeB/FeJ mice for non-clinical efficacy testing, especially for investigating the interaction of lesion pathology and drug effect. Careful use of this model could enhance the bridging of non-clinical results with clinical outcomes.

## Abstract (precis)

**Summary:** C3HeB/FeJ mice develop a wide range of lesion types that alter drug response in a way that might better inform tuberculosis drug development.

## INTRODUCTION

Mice are the animal species most commonly used for evaluating the activity of new and existing drugs against tuberculosis (TB). Although experiments in mice forecast the sterilizing activity of rifampin (RIF) and pyrazinamide (PZA), and the lack of sterilizing activity of other first- and second-line TB drugs, the value of murine models for TB drug development continues to be debated (Grosset, 1978; Mitchison and Chang, 2009). One argument against murine models is that commonly used mouse strains such as BALB/c, C57BL/6 and Swiss mice do not develop such pathological hallmarks of human TB as caseating granulomas and cavitary lesions after infection with *Mycobacterium tuberculosis*. Because C3HeB/FeJ mice were recently found to develop caseation necrosis and occasional cavities after *M. tuberculosis* infection (Kramnik et al., 1998; Driver et al., 2012), they have become a candidate to supplement, or even replace, conventional mouse strains in the non-clinical evaluation of new TB drugs. However, additional comparative studies are needed to evaluate the potential impact of pathological differences on drug efficacy.

The relationship between plasma drug exposures and antibacterial effect in animal models might be affected by differences in lesion microenvironments, the ratio of intracellular to extracellular bacilli, drug penetration into lesions and bacterial burden, which can, in turn, lead to differences in the representation of drug efficacy, with important implications for a given model's utility in forecasting efficacy in humans. For example, the activity of clofazimine is reduced in the caseous lung lesions of C3HeB/FeJ mice compared with BALB/c mice, which do not develop such pathology (Irwin et al., 2014). Another comparative study suggested that PZA has reduced efficacy in C3HeB/FeJ mice compared with BALB/c mice. However, this study used different infection incubation periods for C3HeB/FeJ mice (6 weeks) and BALB/c mice (2 weeks) (Driver et al., 2012). A dose-ranging study comparing RIF and rifapentine (RPT), alone and in combination with first-line drugs, did not demonstrate any significant differences in drug efficacy between C3HeB/FeJ and BALB/c mice (Rosenthal et al., 2012). However, significant inter-individual differences in the presence and extent of caseation was noted among mice of the former strain when treatment was initiated 6 weeks after infection, suggesting that longer infection incubation periods might have been required to observe larger and more uniform necrotic lesions.

In the present study, we sought to determine whether extending the incubation period enables the development of more extensive and more uniform caseous lung lesions and, in turn, alters the treatment response in C3HeB/FeJ mice compared with BALB/c mice. Using three different incubation periods after aerosol infection of both mouse strains, we compared the efficacy of six agents with known anti-TB activity that were selected to test specific hypotheses regarding the interaction between lesion pathology and drug effect. PZA and metronidazole (MTZ) were selected because they require specific conditions [acidic pH and pronounced hypoxia, respectively (Wayne and Sramek, 1994; Zhang et al., 2002)] for anti-TB activity at clinically relevant concentrations and could therefore serve as probes for differences in lesion pH and/or hypoxia, which could cause differential drug effects. For example, portions of necrotic granulomas in C3HeB/FeJ mice might be sufficiently hypoxic for MTZ to be active, as observed in rabbits and macaques but not conventional mouse strains (Via et al., 2008; Driver et al., 2012; Harper et al., 2012). Furthermore, although pus found in abscesses caused by pyogenic bacteria tends to have an acidic pH, what little evidence exists for liquefied caseum of TB lesions suggests a more neutral pH (Koller and Leuthardt, 1934; Weiss et al., 1954), at which PZA would be less effective (Zhang et al., 2002). Because RPT accumulates intracellularly to a greater extent than RIF and is proportionally more potent against intracellular bacilli (Mor et al., 1995), we hypothesized that RPT would be relatively less effective than RIF against the higher proportion of extracellular bacilli in the necrotic lesions of C3HeB/FeJ mice compared with the non-necrotic lesions of BALB/c mice (Rosenthal et al., 2007, 2012). In the same way, sutezolid (PNU) has more potent intracellular activity than linezolid (LZD) and should be relatively less effective than LZD in C3HeB/FeJ mice compared with BALB/c mice (Williams et al., 2009; Zhu et al., 2014).

**TRANSLATIONAL IMPACT**

**Clinical issue**

Tuberculosis (TB) is one of the commonest infectious diseases worldwide and a leading cause of death among persons living with HIV. New drugs to simplify treatment and improve outcomes for drug-resistant forms of TB are desperately needed. Although mice are commonly used as non-clinical efficacy models, most mouse strains do not develop lung pathology similar to human TB, leading to concerns that results of drug treatment in mice might not always predict results in humans. However, when infected with aerosols of *Mycobacterium tuberculosis*, the bacterium that causes TB, C3HeB/FeJ mice develop caseous lung lesions, a type of lesion often seen in individuals with TB. C3HeB/FeJ mice might, therefore, better represent human TB than commonly used mouse strains such as BALB/c mice.

**Results**

Here, the authors investigate whether the use of C3HeB/FeJ mice instead of BALB/c mice influences treatment responses to a panel of TB drugs. They show that, after aerosol infection, C3HeB/FeJ mice develop a range of lesion types (including caseous lesions) that are more consistent with human TB than the lesions that develop in BALB/c mice, and that the development of these lesions alters the response to some TB drugs. For example pyrazinamide had little or no activity in a subset of C3HeB/FeJ mice with large caseous lesions but showed significant bactericidal activity in C3HeB/FeJ mice without such lesions and in BALB/c mice. Other experiments indicate that liquefying caseum in the large caseous lesions is not sufficiently acidic for pyrazinamide to have bactericidal effects and that extending the time between infection and treatment initiation does not affect drug efficacy in C3HeB/FeJ mice.

**Implications and future directions**

Disappointing recent results of long and costly Phase 3 trials have intensified the uncertainty over the predictive accuracy of murine models in TB drug development. Although no other animal model has been convincingly shown to have greater predictive potential, it stands to reason that models that have a close pathological resemblance to human TB are preferable if other considerations (e.g. economics) are largely equal. In this regard, these results suggest that the use of C3HeB/FeJ mice as a complementary or alternative murine model might improve the predictive accuracy of non-clinical efficacy models and improve the success rate of clinical development programs. Further work is needed to compare the predictive accuracy of C3HeB/FeJ mice with that of commonly used mouse strains on the basis of past, present and future clinical trial results.

Portions of the results of this study have been presented previously at the Keystone Symposium on Novel Therapeutic Approaches to Tuberculosis (Abstract No. 235, Keystone, CO, April 2014), the International Workshop on the Clinical Pharmacology of Tuberculosis Drugs (Abstract No. 12, Washington, DC, September 2014), and the Interscience Conference on Antimicrobial Agents and Chemotherapy (Abstract No. A-20, Washington, DC, September 2014).

## RESULTS

### Lung CFU counts upon implantation and initiation of treatment

A separate aerosol infection was performed for each of the three arms of incubation (6, 10 and 14 weeks). Mean lung colony-forming unit (CFU) counts determined on the day after aerosol infection and at the initiation of treatment (D0) are provided in Table 1. The mean infectious doses implanted were 1.38 and 2.15 log_10_ CFU/lung for C3HeB/FeJ and BALB/c, respectively, leading to D0 counts between 6.7 and 7.7 log_10_ CFU/lung for C3HeB/FeJ mice and 6.2 and 6.8 for BALB/c mice. The differences between mean CFU counts in the three arms of incubation (6, 10 or 14 weeks) were not statistically significant (*P*=0.08) for C3HeB/FeJ mice (probably owing to lack of power), but were statistically different (*P*=0.03) for BALB/c mice.

**Table 1.**
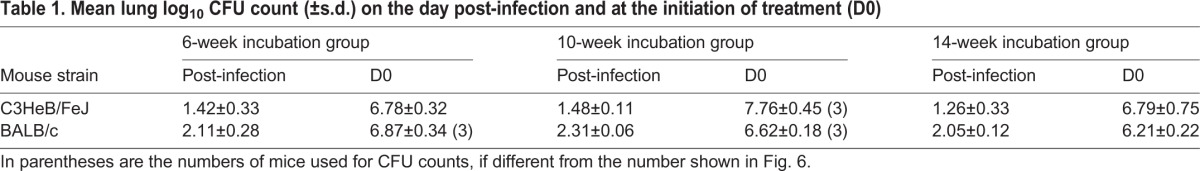
**Mean lung log_10_ CFU count (±s.d.) on the day post-infection and at the initiation of treatment (D0)**

### Mortality

**Fig. 1.**
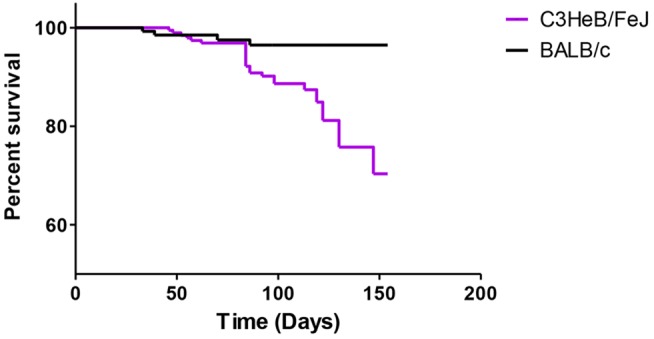
**Aggregate survival of untreated and treated mice from the day of infection.** Mice that were sacrificed at predetermined time points were censored. A higher rate of death was observed in C3HeB/FeJ mice compared with BALB/c mice.

**Fig. 2.**
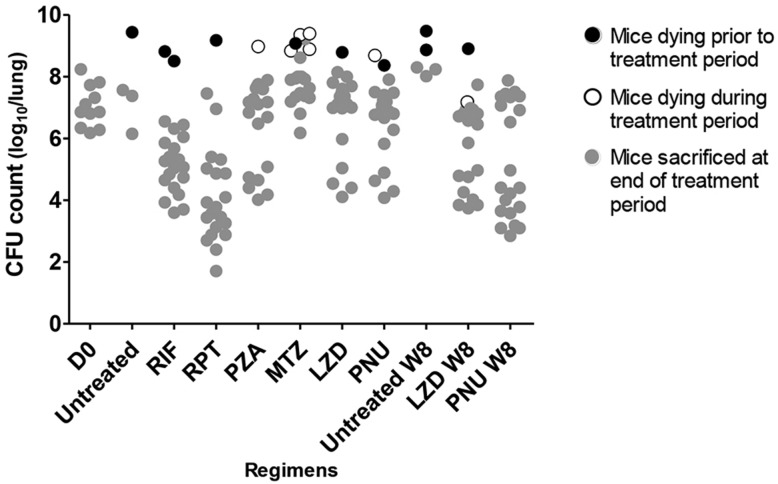
**Lung CFU counts among C3HeB/FeJ mice.** The treatment period was 4 weeks except in groups designated by ‘W8’, which received 8 weeks of treatment. Results from each incubation arm are combined. D0, initiation of treatment; RIF, rifampin; RPT, rifapentine; PZA, pyrazinamide; MTZ, metronidazole; LZD, linezolid; PNU, sutezolid.

We observed a higher rate of death in C3HeB/FeJ mice compared with BALB/c mice (29.6% vs. 3.6%, respectively; *P*<0.01) (Fig. 1). For the 17 (of 26) premature deaths among C3HeB/FeJ mice for which lung CFU counts are available, they are presented in Fig. 2. Of these 17 premature deaths, eight occurred before initiation of treatment and nine after. The greatest number of deaths occurred among MTZ-treated C3HeB/FeJ mice, of which six of 21 mice allocated to treatment died, with five deaths occurring during treatment. All of these deaths were attributed to progressive TB. Three additional MTZ-treated mice in the 14-week incubation group were euthanized 4 days prior to the planned sacrifice date as a precaution against losing the mice over the weekend before the sacrifice date due to their ill status, but were not counted as deaths during treatment. Taking all deaths into consideration, the rate of death seemed to be constant during the course of the study, beginning about 7 weeks post-infection, confirming that C3HeB/FeJ mice are more susceptible to TB but, under the conditions studied, either progress at different rates or progress variably owing to early, stochastic events that set severe pathology in motion. The duration of the incubation period had no impact on this death rate (*P*=0.11; data not shown).

### Assessment of treatment efficacy

**Fig. 3.**
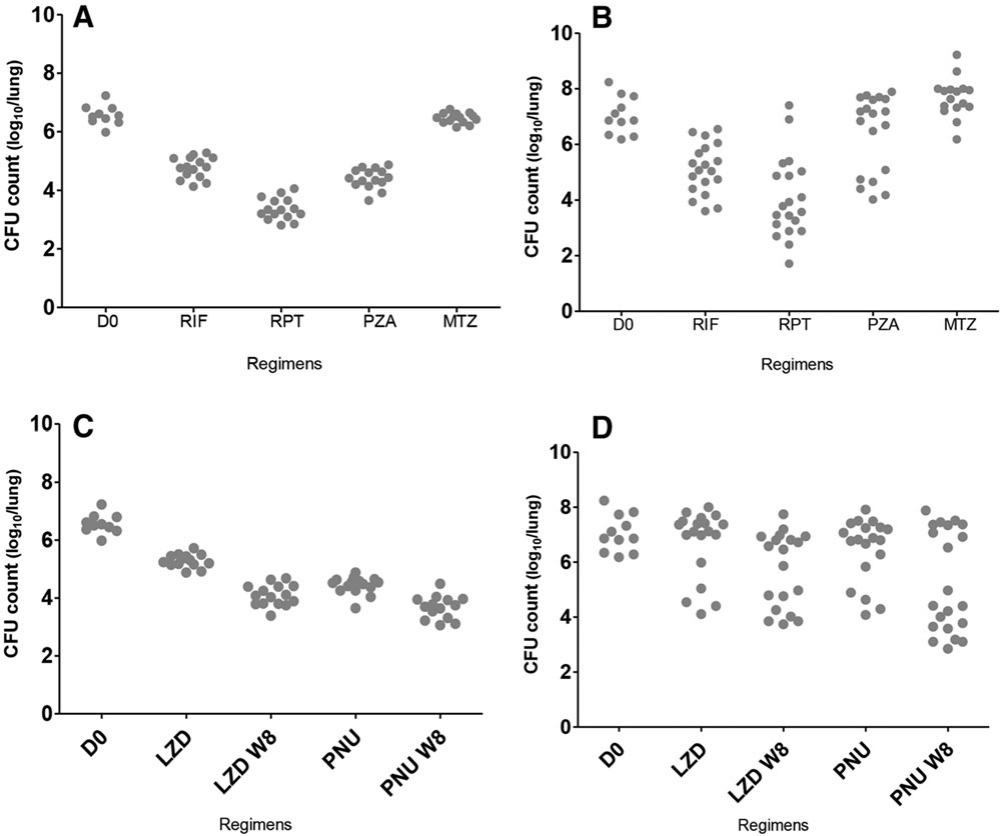
**Individual lung CFU distribution by mouse strain.** CFU distribution in BALB/c mice is shown on the left (A,C), and in C3HeB/FeJ mice on the right (B,D). Oxazolidinones are presented separately in panels C and D. RIF, rifampicin; RPT, rifapentine; PZA, pyrazinamide; MTZ, metronidazole; LZD, linezolid; PNU, sutezolid; W8, results after 8 weeks of treatment. D0 is the day of treatment initiation.

As expected, the rifamycins, oxazolidinones and PZA were clearly active in BALB/c mice, whereas MTZ was not. The Δlog_10_ CFU [i.e. mean D0 CFU count – mean W4 (4 weeks post-treatment) CFU count] values were 1.76 for RIF, 3.20 for RPT, 2.16 for PZA, 1.28 for LZD, 2.13 for PNU and 0.10 for MTZ. The Δlog_10_ CFU was even greater after 2 months of treatment for both LZD (2.47) and PNU (2.89). All mice responded similarly to treatment regardless of the incubation arm (Fig. 3A,C; supplementary material Fig. S1A,C). The linear analysis model showed an interaction between drugs and incubation period at both W4 and W8 (*P*=0.01 and 0.046, respectively), rendering the comparisons between the incubation periods meaningless. However, for any incubation period the pairwise comparisons showed that PNU was statistically more effective than LZD, and RPT was more effective than RIF.

Lung CFU counts among C3HeB/FeJ mice revealed greater variability, which reduced the power to discriminate differences in treatment efficacy. Only RIF and RPT demonstrated convincing bactericidal activity (Fig. 3B,D), with Δlog_10_ CFU values (1.94 for RIF and 3.00 for RPT) similar to those in BALB/c mice. MTZ was ineffective in C3HeB/FeJ mice, as in BALB/c. However, unlike what was observed in BALB/c mice, PZA, PNU and LZD demonstrated dichotomous effects, in which treated mice segregated into two distinct response groups, with one group exhibiting bactericidal effects comparable to those in BALB/c mice and the other demonstrating little or no reduction in CFU (Fig. 3B,D). This dichotomous activity occurred independently of both incubation period and treatment duration (supplementary material Fig. S1B,D). Acknowledging that the observed heterogeneity undermines the power of statistical comparisons, the linear model revealed no interaction between treatment and incubation period (*P*=0.16 and 0.13 at W4 and W8, respectively), suggesting that the incubation period did not significantly affect drug efficacy. Pairwise comparisons did not reveal statistically significant differences in effect size between RIF and RPT (*P*=0.17) or between PNU and LZD (*P*=1.00).

### Assessment of drug resistance

To confirm that heterogeneity in treatment response was not due to drug resistance, colonies from the lungs of seven mice with the highest CFU counts [two C3HeB/FeJ mice receiving RPT and five mice receiving PNU (four C3HeB/FeJ mice and 1 BALB/c mouse)] were replated on drug-containing media. Resistant mutants were observed only in the BALB/c mouse in the 14-week incubation group treated for 8 weeks with PNU, but the resistant colonies represented <1% of the total CFU.

### Pathology

**Fig. 4.**
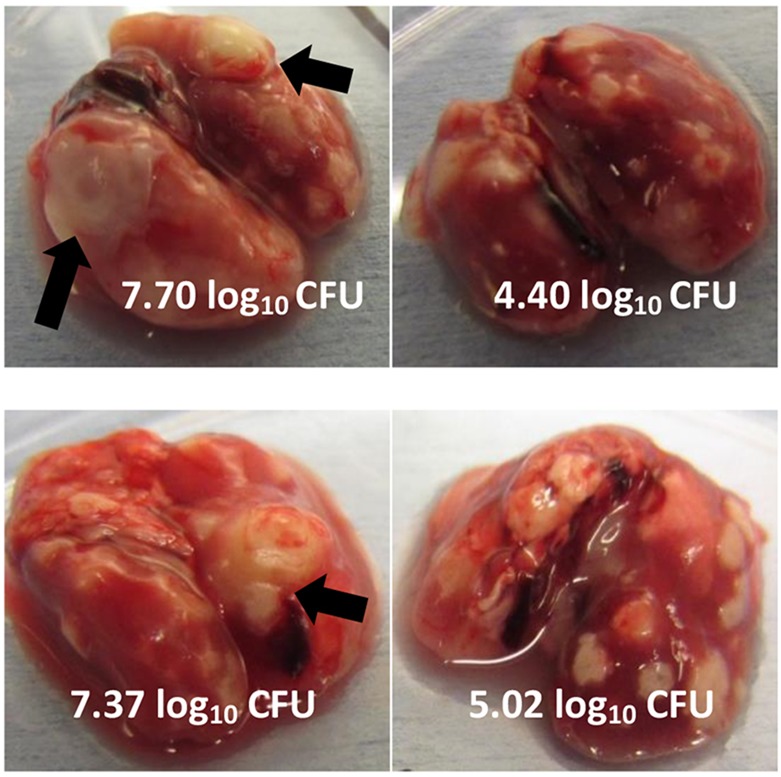
**Examples of gross pathology and correlation with lung CFU counts from mice in the 14-week incubation arm following treatment with pyrazinamide or linezolid.** PZA, upper panels; LZD, lower panels. Arrows show large, expansile tubercular lesions in the lungs with the highest CFU counts.

We hypothesized that the heterogeneity in CFU counts across treatment groups was determined by differences in the extent of lung pathology at the time of treatment initiation. Inspection of the lungs at the time of necropsy demonstrated large, expansile tubercular lesions in the lungs of PZA- and oxazolidinone-treated mice segregating in the high-CFU-burden group of C3HeB/FeJ mice, and small discrete lesions similar to those in BALB/c mice in the group with a low CFU burden (Fig. 4).

**Fig. 5.**
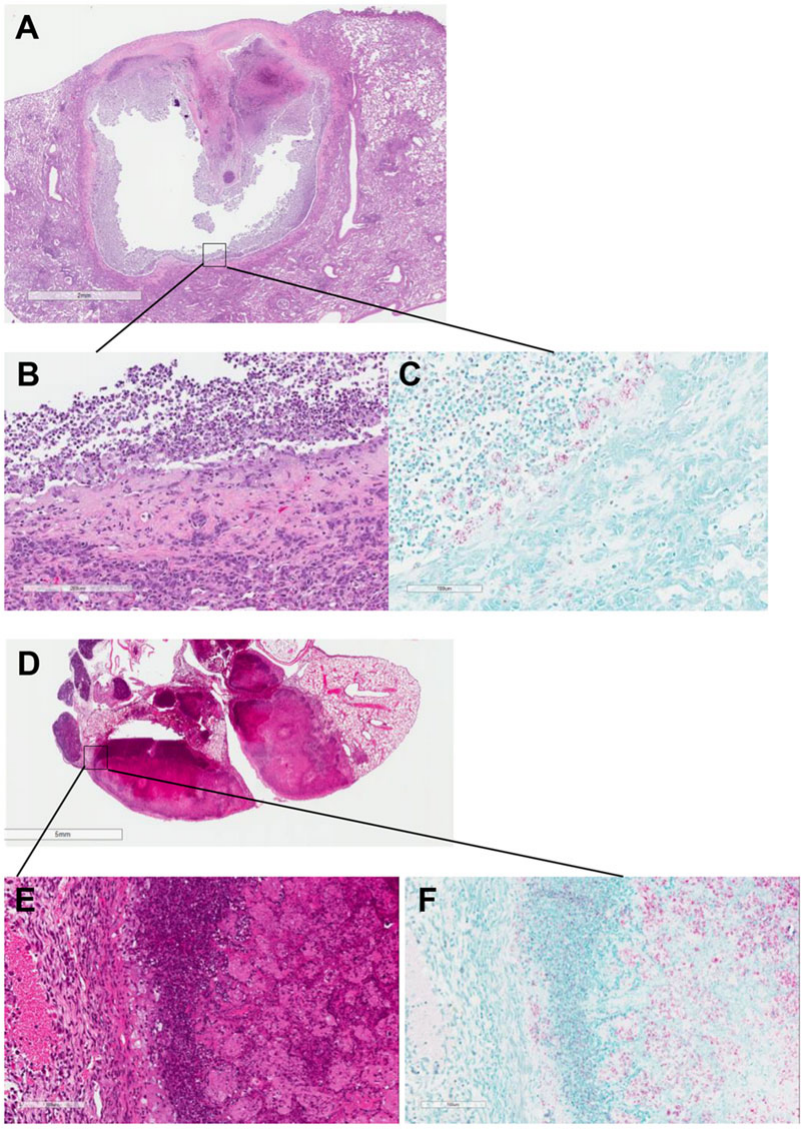
**Histopathology of caseous lesions in untreated C3HeB/FeJ mice at different incubation times.** (A) Lung cavity formed in a C3HeB/FeJ mouse after 26 weeks of incubation. (B,C) Cavity wall with H&E (B) and acid-fast bacilli (AFB; C) staining. (D) Sick mouse after 8 weeks of incubation. (E,F) Granuloma wall with monocyte infiltration and foamy macrophages with H&E (E) and AFB (F) staining. Scale bars: 2 mm (A); 200 μm (B); 100 μm (C,E,F); 5 mm (D).

Histopathological analysis of the lungs confirmed the previously described differences between BALB/c and C3HeB/FeJ mice, i.e. the development of caseating granulomas and caseous pneumonia in the latter strain (Davis et al., 2009; Driver et al., 2012; Marzo et al., 2014). Solid caseous granulomas showed neutrophil-predominant inflammatory cell infiltrates at the interface between the foamy macrophage layer and caseum, with differing degrees of neutrophilic infiltration into the otherwise acellular caseum (Fig. 5). Although occasional cavitary lesions were described by Driver et al. (2012), we present here further clear-cut histopathological evidence of a chronic cavity with a fibrotic capsule and mixed inflammatory infiltration with numerous neutrophils, especially in the liquefied caseum lining the cavity wall, where many neutrophils harbored acid-fast bacilli (Fig. 5). BALB/c mice demonstrated the typical mononuclear cell aggregates containing foamy macrophages (Rosenthal et al., 2012), and longer incubation periods led to an increase in infiltration, first with lymphocytes then with foamy macrophages and fibrosis. Remarkable heterogeneity in the presentation of disease in C3HeB/FeJ mice was observed. Whereas some mice showed very similar, if not indistinguishable, pathology compared with BALB/c mice, even after 22 weeks of incubation, other C3HeB/FeJ mice developed a massive burden of caseation necrosis within the first 8 weeks of incubation (supplementary material Fig. S2).

### pH measurement

Because the anti-tuberculosis activity of PZA is pH-dependent and might only be expected to occur at plasma concentrations achievable with the 150 mg/kg dose, when the bacilli reside in an acidic milieu, we hypothesized that the poor activity of PZA in large caseous lesions is the result of insufficiently acidic conditions. Thirteen different large lesions from five different mice were selected to measure the pH of liquefied caseum therein. An average pH of 7.39±0.096 (range 7.19-7.54) was observed, similar to that of the adjacent normal-appearing lung and that of uninfected mouse lung (7.38±0.082).

## DISCUSSION

After *M. tuberculosis* infection, C3HeB/FeJ mice, unlike the other strains currently used to model TB, develop caseous lesions, as seen in active TB in humans; C3HeB/FeJ mice are therefore appealing as a model to complement, or even replace, other commonly used mouse strains for non-clinical drug efficacy testing. In the present study, the activity of a panel of anti-TB drugs was compared in C3HeB/FeJ and BALB/c mice to investigate whether differences in lung pathology influence drug efficacy (e.g. owing to alterations in lesion microenvironment, intracellular versus extracellular bacterial ratios and/or drug penetration). The results confirm and extend our (A.L.) prior work by showing that necrotic lesions of C3HeB/FeJ mice are likely not sufficiently hypoxic for MTZ to work even with an extended incubation period (Driver et al., 2012). This could be related to the smaller size of the necrotic lesions and smaller hypoxic gradient in C3HeB/FeJ mice relative to rabbits and macaques, in which anti-mycobacterial activity of MTZ has been suggested (Via et al., 2008). The activity of the rifamycins RIF and RPT in C3HeB/FeJ mice was also generally similar to that observed in BALB/c mice, albeit with greater inter-mouse variability, which limited statistical power. Despite the suggestion of greater potency of RPT over RIF in C3HeB/FeJ mice on average, we note that two RPT-treated mice had significantly higher lung CFU counts than other RPT-treated mice, and also higher than those of any RIF-treated mouse; therefore, we cannot exclude the possibility that RPT is not more potent than RIF in mice with the largest caseous lesions and carrying the highest burdens of bacteria, a result that could reflect reduced RPT diffusion, accumulation or activity in caseous lesions. Results from a recent Phase 2 trial have suggested that experiments in BALB/c mice might have underestimated the RPT dose that is equipotent to the standard RIF dose in humans (Dorman et al., 2012). Further dose-ranging comparisons of RIF and RPT in severely affected C3HeB/FeJ mice are needed to determine whether these mice can better represent comparative rifamycin activity in patients with active pulmonary TB than currently used strains.

As recently shown for clofazimine, the discrepant activity of PZA in BALB/c and C3HeB/FeJ mice highlights the potential importance of lesion-specific characteristics in the efficacy of some anti-tuberculosis drugs (Irwin et al., 2014). Although reduced penetration of PZA into large necrotic lesions was not excluded in this study, PZA has been shown to diffuse uniformly through caseous lesions of infected rabbits (Dartois, 2014). The activity of PZA is strongly pH-dependent. The expected minimal inhibitory concentration (MIC) of PZA at the neutral pH of 7.4 observed in the liquefied caseum is >1000 µg/ml, far above concentrations achievable in plasma and epithelial lining fluid (Conte et al., 1999; Zhang et al., 2002). On the other hand, bacilli in the phagosomes of activated macrophages, which predominate in a chronic infection model in BALB/c mice and in C3HeB/FeJ mice without large caseous lesions, can experience pH levels below 5, where the expected PZA MIC would be 10 µg/ml or less (Zhang et al., 2002; Vandal et al., 2008). Thus, differences in the lesion microenvironment encountered by bacilli in large caseous lesions as compared to those inside active macrophages might explain the differences in PZA efficacy within C3HeB/FeJ mice with different lesion types, and between C3HeB/FeJ and BALB/c mice. Although caseum has often been assumed to be acidic owing to its necrotic origins, there is little direct evidence to support this assumption. Experiments conducted over 60 years ago in rabbits infected with *Mycobacterium bovis* suggest that, whereas the centers of early tubercles have a pH of 6.4-6.7, the liquefied contents of softening tubercles later in the disease have a pH of 7.3-7.4 (Weiss et al., 1954), consistent with our results. To our knowledge, our study is the first to report the pH of liquefied caseum in lesions produced by *M. tuberculosis* infection in any non-human species. pH measurements in other non-clinical models of TB are now required to confirm our results.

The comparative pathological and microbiological assessments performed here indicate that use of C3HeB/FeJ mice brings additional dimensions to the assessment of drug efficacy in murine models. In particular, the advanced necrotic lesions harbor large numbers of extracellular bacilli and might cavitate with features consistent with those described in unfavorable cavitary lesions in humans (Canetti, 1955). Therefore, C3HeB/FeJ mice could be an excellent model to study the interaction between lesion characteristics, drug exposures at the infection site and treatment response. Better understanding of these relationships could improve the ability of drug efficacy studies in mice to represent patients with the most severe TB disease, as characterized by cavitary disease and/or high bacillary burden in sputum, who are at increased risk of relapsing when treatment duration is too short (Benator et al., 2002; Merle et al., 2014). However, the heterogeneity in the timing and extent of disease progression in C3HeB/FeJ mice presents a challenge for routine use as a model for evaluating drug efficacy.

Previously published studies have described large inter-individual variation in lung CFU counts and lung pathology in C3HeB/FeJ mice after low-dose aerosol infection (Driver et al., 2012; Rosenthal et al., 2012; Irwin et al., 2014), which reduces the power for discriminating differences between treatment groups. The current study was conceived, in part, to investigate whether extending the incubation period would help to reduce such heterogeneity. In fact, extending the incubation period did not significantly reduce the heterogeneity in lung CFU counts and pathology. On the contrary, we observed decidedly more heterogeneity in disease progression than we previously observed in C3HeB/FeJ mice (Driver et al., 2012; Rosenthal et al., 2012; Irwin et al., 2014). That this heterogeneity happened independently of the incubation arm and some mice did not develop large necrotic lesions despite incubation for 26 weeks whereas other untreated animals succumbed to the disease suggests that the factors or events responsible for setting such disease progression in motion likely occur in the first few weeks of incubation. The use of PZA and oxazolidinones likely only exacerbated such differences in disease progression and resultant bacterial burden present at initiation of treatment by proving quite bactericidal in mice without large caseous lesions, where the lower pH and higher ratio of intracellular to extracellular bacilli might favor drug activity, but only weakly bactericidal in mice with advanced disease. This dichotomous activity produced two distinct treatment response profiles, one much like the response observed in BALB/c mice and another that was distinctly worse. In the latter case, the poor response occurs in large necrotic lesions with very high bacterial burdens and might mask bactericidal effects in smaller, largely cellular lesions occurring in the same animals. The limited bactericidal activity observed in severely affected mice might be more consistent with the limited early bactericidal activity observed with these drugs in humans (Jindani et al., 1980; Dietze et al., 2008; Everitt et al., 2013; Wallis et al., 2014). Prior work might not have revealed the same degree of heterogeneity because of the use of shorter incubation periods, use of more potent drugs or combination therapies, or loss of the most severely affected mice before treatment onset (Driver et al., 2012; Rosenthal et al., 2012; Irwin et al., 2014).

Although the observed heterogeneity reduced the power to discriminate differences between the rifamycins and between the oxazolidinones in C3HeB/FeJ mice, it offered valuable opportunities to better understand the influence of lesion type and microenvironment on drug action. For example, the differential activity of PZA based on lesion type in C3HeB/FeJ mice deepens our understanding of where this unique pH-dependent drug exerts its sterilizing activity in TB patients in a way that no other animal model has. If the neutral pH of caseum in C3HeB/FeJ mice can be validated in caseous lesions of humans with progressive TB, this result alone could justify the use of C3HeB/FeJ mice in the non-clinical evaluation of promising TB drug and regimen candidates, because PZA is not the only TB drug whose activity is influenced by pH (Moellering, 1983; Burian et al., 2012). To improve the utility of C3HeB/FeJ mice for more routine drug efficacy testing, it would be useful to develop either a means of creating more predictable and uniform disease progression and/or to develop more effective ways of tracking the treatment response in individual mice to minimize the negative impact of heterogeneity on the power to determine differences between treatments. The former objective will likely require a better understanding of the factors responsible for differences in disease progression in these inbred mice. The latter might be achievable through the serial assessment of biomarkers, including emerging imaging or molecular diagnostic technologies.

In conclusion, our study highlights the potential of C3HeB/FeJ mice as a more pathologically relevant murine model of progressive TB, adding value to non-clinical drug efficacy testing. C3HeB/FeJ mice can develop a range of pathological features, including caseation, liquefactive necrosis and cavitation, which better resemble the pathology of human TB than the changes observed in other mouse strains (Irwin et al., 2015). As demonstrated for CFZ (Irwin et al., 2014), PZA and the oxazolidinones, the response to some TB drugs differs by lesion type, making C3HeB/FeJ mice an excellent model to study the interaction between lesion characteristics (e.g. microenvironmental conditions), drug exposures at the infection site and treatment response. Better understanding of these relationships should improve the representation of drug efficacy in mice.

## MATERIALS AND METHODS

### Mycobacterial strains

*M. tuberculosis* H37Rv was used as a frozen stock prepared from a log-phase culture in Middlebrook 7H9 broth after mouse passage and was diluted in 7H9 broth before infection (Rosenthal et al., 2012).

### Drugs

RIF, RPT, PNU, LZD and PZA were obtained and formulated for oral administration as previously described (Williams et al., 2012). MTZ was obtained from Sigma Chemical Co. (St Louis, MO) and prepared in pure water as described by Chew et al. (2012).

### Aerosol infection with *M. tuberculosis*

All animal procedures were approved by the Animal Care and Use Committee of Johns Hopkins University. Six- to nine-week-old female BALB/c mice (Charles River, Wilmington, MA) and C3HeB/FeJ mice (Jackson, Bar Harbor, ME) were used. Mice were aerosol-infected using the Inhalation Exposure System (Glas-Col, Terre Haute, IN) using 40-fold and 300-fold dilutions of a titered frozen stock of *M. tuberculosis* H37Rv, for BALB/c and C3HeB/FeJ mice, respectively. One day after infection, four mice from each of two aerosol runs were humanely killed to determine the number of bacteria implanted in the lungs.

### Study design

**Fig. 6.**
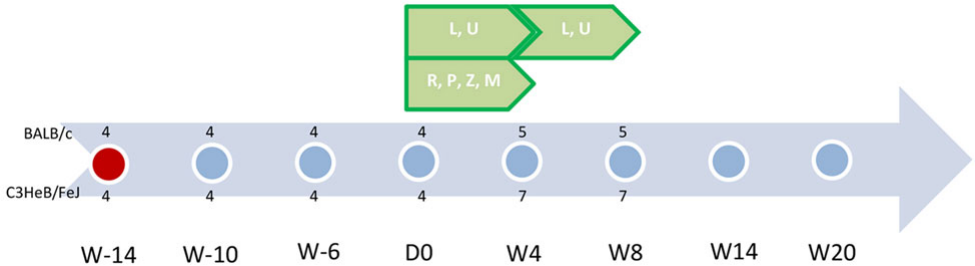
**Scheme of experiment.** Abbreviations: R, rifampin; P, rifapentine; Z, pyrazinamide; L, linezolid; U, sutezolid (PNU-100480); M, metronidazole. Each dot represents a time point for pathology assessment (two untreated mice were included per strain); red dot is the time of infection. Green pentagons represent treatment administered and its duration. Numbers represent the number of mice killed at each time point for each treatment group (above the dot for BALB/c mice and below the dot for C3HeB/FeJ mice). W-14 is 14 weeks prior to initiation of treatment. D0 indicates the day of treatment initiation. W20 is 20 weeks following treatment initiation. This figure represents the scheme as applied to the 14-week incubation cohort. A similar scheme was carried out for the 10-week and 6-week incubation cohorts.

The experimental schemes are detailed in Fig. 6. Three different incubation periods were applied before treatment initiation (D0): 6 weeks, 10 weeks and 14 weeks. Each incubation group was infected separately (implantation) using the same frozen stock. All incubation groups were treated for 4 weeks with the following drugs (dose, in mg/kg body weight): RIF (10), RPT (10), PNU (50), LZD (100), PZA (150) and MTZ (500). Additional mice were treated for a total of 8 weeks with PNU and LZD. Treatment periods for each incubation group overlapped in time. Untreated mice previously allocated for pathological assessment (described below) were sacrificed every 4-6 weeks.

### Assessment of treatment efficacy

Treatment efficacy was assessed on the basis of lung CFU counts during treatment. Serial dilutions of whole-lung homogenates were performed on selective Middlebrook 7H11 agar plates enriched with 10% oleic acid, albumin, dextrose and catalase complex (OADC) (Becton Dickinson, Franklin Lakes, NJ). Plates were incubated for 6-8 weeks at 37°C before determining final CFU counts.

### Assessment of drug resistance

The selection of drug-resistant mutants was assessed only in a subset of mice with unexpectedly high CFU counts, i.e. >2 log_10_/ml difference than the mean of the mice in the same arm. Colonies were scraped and collected from agar plates and suspended by bead-beating (0.5-mm sterile beads) in 2.5 ml of phosphate buffered saline (PBS). Quantitative cultures were performed on selective 7H11 agar with 10% OADC with or without 1 µg/ml of RIF, PNU or the principal metabolite of PNU, PNU-101603.

### Pathology

For mice dedicated to histopathology, lungs underwent *in situ* fixation using methods adapted from Braber et al. (2010). Mice were killed by an overdose of isoflurane (Baxter, Deerfield, IL). The animals were exsanguinated by cutting the caudal vena cava to prevent the flow of blood into the bases of the lungs. A cannula was inserted into the trachea and fixed with a ligature. After opening of the thorax lungs were inflated *in situ* via the cannula by injection of 10 ml of 10% formalin (Sigma-Aldrich, St Louis, MO) then by infusion for 5 min at a constant fluid pressure of 35 cm. After the trachea was tied off with a ligature, we removed the lungs and placed them in a 50 ml conical tube containing 10% formalin. After 48 h, the 10% formalin was replaced by 70% ethanol until shipment when the lungs were transferred to PBS.

Because BALB/c mice received a higher infectious dose and developed more homogenous and uniformly distributed lung lesions than did C3HeB/FeJ mice, only the right lung was sampled for pathology. The injection of 10% formalin was then done after both ligature of the left bronchus and removal of the left lung for CFU-count assessment.

Tissue sections were embedded in paraffin and cut to 5 μm thickness on a microtome. Subsequent tissue sections were mounted on glass slides, deparaffinized and stained with hematoxylin and eosin (H&E). Sections were scanned and visualized using Aperia Imagescope software (Leica Biosystems, Nussloch, Germany), allowing a high variety of magnification.

### Assessment of pH of the lesions

The pH of liquefied caseum was measured in selected lesions of more than 3-mm diameter in C3HeB/FeJ mice with a 16 G needle tip micro-pH comb electrode (Thermo Scientific Orion, Chelmsford, MA) and a benchtop pH meter (Mettler Toledo FE20, Business Unit Analytical, Schwerzenbach, Switzerland). Immediately after sacrifice and dissection to expose the lung, the pH probe was inserted directly into the lesion *in situ* to measure the pH of the liquefied material.

### Statistical analysis

CFU counts (*x*) were log-transformed as log_10_(*x*+1) before analysis. To test whether the incubation time affected the drug efficacy within each mouse strain, we performed a general linear model analysis of a 2-factor model (drug and incubation) with interaction term and robust standard errors for each time point (W4 and W8). To compare drug effects within each mouse strain, we performed pairwise comparisons, comparing drugs and comparing drug-incubation pairs. A *P*-value of ≤0.05 was considered significant. The Bonferroni correction was used to adjust for multiple comparisons. The differences between mouse strains were descriptively analyzed.

## Supplementary Material

Supplementary Material
