# Supplementary material for: Heterogeneous disease progression and treatment response in a C3HeB/FeJ mouse model of tuberculosis
Source: Dis Model Mech. 2015 Jun 1;8(6):603–10. doi: 10.1242/dmm.019513 (PMC4457036; doi:10.1242/dmm.019513)
Supplement: Supplementary Material [file supp_8_6_603__index.html]

Heterogeneous disease progression and treatment response in a C3HeB/FeJ mouse model of tuberculosis — Supplementary Material 

# Heterogeneous disease progression and treatment response in a C3HeB/FeJ mouse model of tuberculosis

## DMM019513 Supplementary Material

**Files in this Data Supplement:**

- **Supplementary Material**
