# Supplementary material for: Heterogeneous disease progression and treatment response in a C3HeB/FeJ mouse model of tuberculosis
Source: Dis Model Mech. 2015 Jun 1;8(6):603–10. doi: 10.1242/dmm.019513 (PMC4457036; doi:10.1242/dmm.019513)
Supplement: Supplementary Material [file supp_8.6.603_DMM019513.pdf]

Supplementary files

Figure S1. Individual lung CFU distribution by mouse strain and incubation arm.

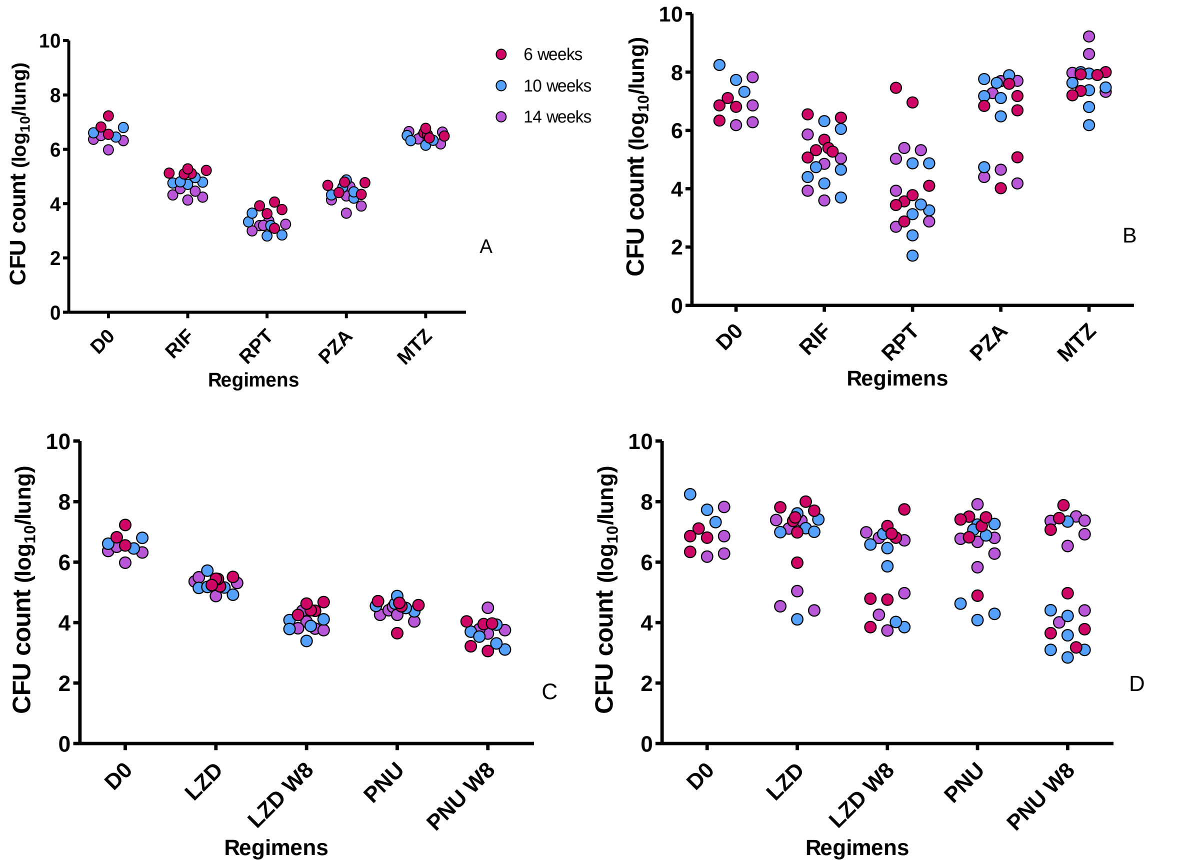

Legend: Individual lung CFU distribution by incubation arm and mouse strain (in BALB/c in panels A and C, in C3HeB/FeJ in panels B and D). Oxazolidinones are presented separately in panels C and D. RIF=rifampicin, RPT=rifapentine, PZA=pyrazinamide, MTZ=metronidazole, LZD=linezolid, PNU=sutezolid. W8 = results after 8 weeks of treatment. D0 is the day of treatment initiation.

Figure S2. Comparison of lung histology between BALB/c and C3HeB/FeJ mice at given incubation times.

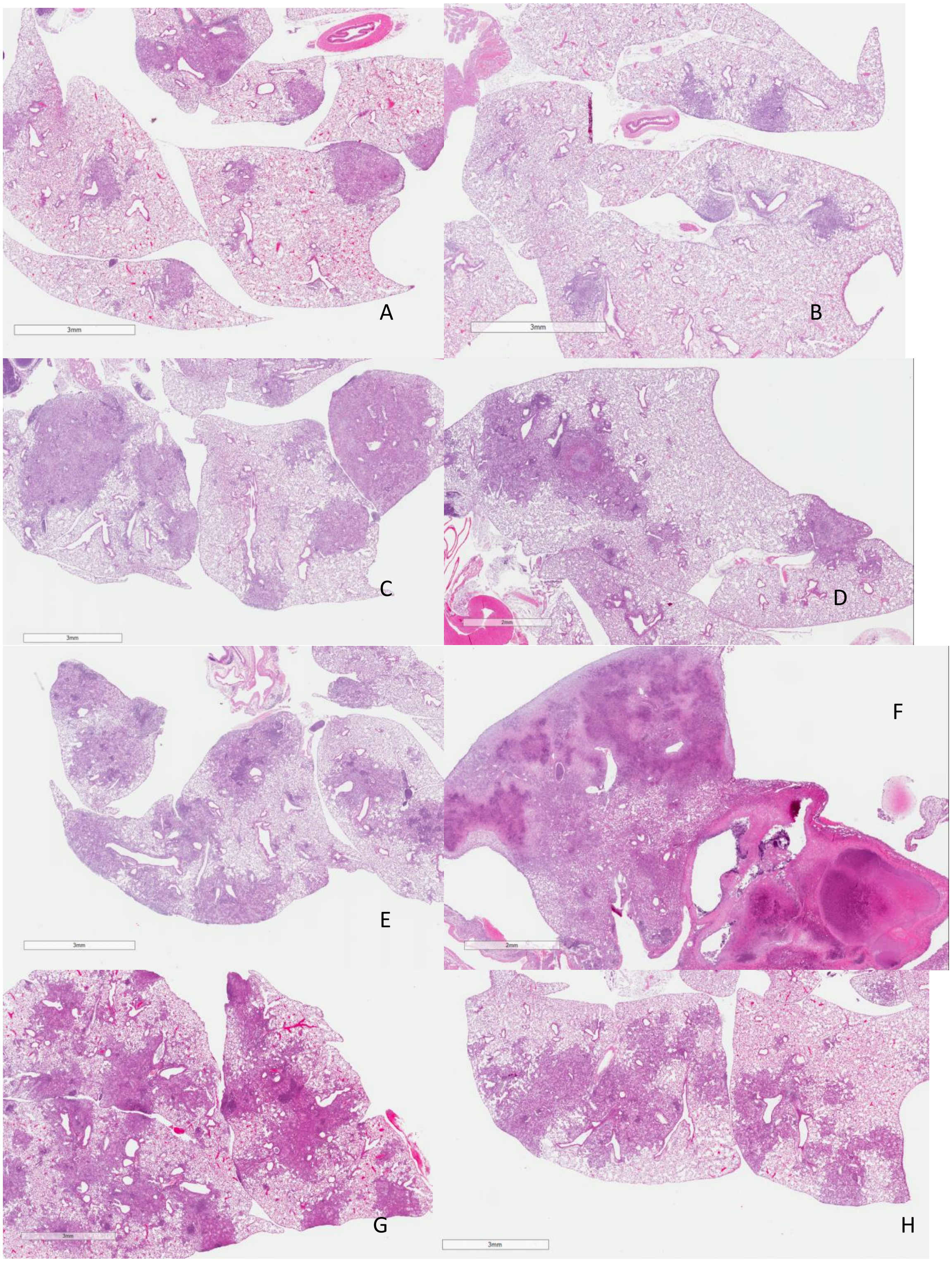

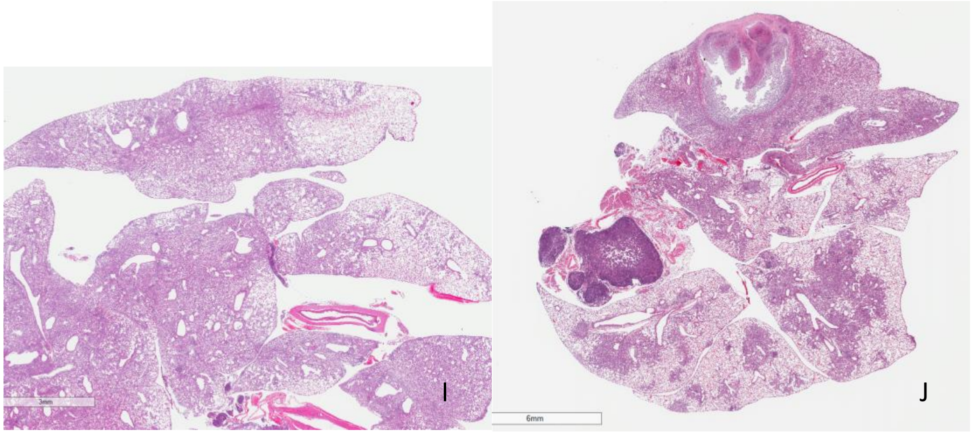

Legend:

Left panel BALB/c mice; Right panel C3HeB/FeJ mice

A-B: 4 weeks of incubation, C-D: 8 weeks, E-F: 14 weeks, G-H: 18 weeks, I-J: 26 weeks

Mice in F and G were sick at the time of sacrifice.
